# Editorial: Initiatives to raise young people's interest and participation in STEM

**DOI:** 10.3389/fpsyg.2023.1151715

**Published:** 2023-02-16

**Authors:** Milagros Sáinz, Katja Upadyaya, Sergi Fàbregues

**Affiliations:** ^1^Internet Interdisciplinary Institute, Universitat Oberta de Catalunya, Barcelona, Spain; ^2^Minds Hub Research Group, Faculty of Educational Sciences, University of Helsinki, Helsinki, Finland; ^3^Department of Psychology and Education, Universitat Oberta de Catalunya, Barcelona, Spain

**Keywords:** interest, participation, gender, Science, Technology, Engineering, and Mathematics (STEM), initiatives

The low share of women in Science, Technology, Engineering, and Mathematics (STEM) is a complex global phenomenon that requires further investigation, since it affects millions of women and girls worldwide. A broad range of interventions, based on a diversity of disciplinary, theoretical, and methodological approaches, have been conducted in different countries and contexts to encourage the participation of young women in various STEM disciplines, especially in those where women remain dramatically underrepresented, such as engineering and physical science. These interventions provide practitioners and policymakers with best practices to tackle the gender gap in STEM careers and professions, promote girls' positive attitudes towards scientific and technological fields, and identify barriers which stand in the way of higher female achievement in science and technology subjects, among other actions. To ensure that these interventions are effective, feasible, and well accepted by their participants, intervention studies grounded in quantitative, qualitative, and mixed methods have been developed.

In the present Research Topic, a variety of aspects related to the under-representation of women in STEM have been addressed with a group of 11 high-quality papers related to gender-based intervention studies. These papers include rigorous empirical studies, methodological papers, and systematic reviews describing initiatives or programs to overcome the gender gap in the STEM educational and career pathways through the following aspects:

- Focusing on research questions and/or objectives related to the gender gap in access and progression of STEM education.
- Drawing on one or more theoretical approaches (i.e., person-environment fit theory, RIASEC model of vocational interests, expectancies and values, social role theory, project-based learning principles, etc.).
- Addressing different stages of educational pathways, including primary, secondary, and higher STEM education.
- Using various methodological approaches to design and evaluate their implementation and effectiveness.
- Discussing the sustainability and long-term effects of the interventions.
- Treating the intersection of gender and other factors, such as areas of study, country of origin, family socioeconomic status, and attained educational level.

The first paper “*Impact of interest congruent on study outcomes”* tackles how social and aspirational congruence interest of a group of German university students are related to students' persistence, performance, and satisfaction in six different study areas, including STEM (Ertl et al.).

In the second paper “*I am done with this. Women dropping out of engineering majors*” the authors conducted a qualitative study with a group of Spanish engineering students, where the main factors (i.e., the influence of stereotypes, lack of role models, excessive academic workload or a hostile class environment) pushing women to drop out of engineering education were identified (González-Pérez et al.).

The third paper entitled “*Girls get Wise. A programming model for engaging girls in STEM*” describes the features and evaluation process of a long-term Canadian university-based program aimed at engaging girls in STEM. Through the use of hands-on interactive STEM activities, this program provides an opportunity for young women to showcase their talents and excitement for science-based topics (Franz-Odendaal and Marchand et al.).

The paper “*On the Design and Validation of Assessing Tools for Measuring the Impact of Programs Promoting STEM Vocations*” addresses the design and validation of an instrument to evaluate how an informal learning initiative developed in Spain promotes Science, Technology, Engineering, and Mathematics STEM vocations among secondary students, their families (parents), and secondary teachers (Herce-Palomares et al.).

The paper “*Perception of work in the IT sector among men and women—A comparison between IT students and IT professionals*” examines gender differences in goal congruence, sense of belonging, and self-efficacy in IT among a group of Polish IT and non-IT workers as well as university students (Pyrkosz-Pacyna et al.).

In the paper “*Interventions to increase young people's interest in STEM. A scoping review*” the authors examine the main characteristics and effectiveness of intervention studies aiming at encouraging secondary school students' interest in STEM over the past 20 years, with a particular focus on female students. Twenty-five studies were also identified as best practices for their design and evaluation characteristics (Sáinz et al.).

The paper “*Associations between adolescent students' multiple domain task value-cost profiles and STEM aspirations*” examines the task value and cost profiles of Finnish middle school students in association with STEM aspirations, and investigates gender differences, using latent transition analysis as a methodological approach (Vinni-Laakso et al.).

The paper “*Gender biases in the training methods of affective computing: Redesign and validation of the Self-Assessment Manikin in measuring emotions via audiovisual clips*” analyzes the development and experimental testing of a graphic design tool for the labeling of emotions free of gender biases (Sainz-de-Baranda Andujar et al.).

The paper “*Gendered difference in motivational profiles, achievement, and STEM aspiration of elementary school students*” uses latent transition analysis to look into gender differences in motivation profiles and their influence on achievement and STEM aspirations over time in a sample of Finnish elementary students (Olive et al.).

The paper “*Intervention initiatives to raise young people's interest and participation in STEM*” examines, using two interventions developed with randomized control trials, how to increase science interest and participation in a group of elementary and secondary school students in the United States (Schneider et al.).

Finally, the paper entitled “*Use of mixed methods research in intervention studies to increase young people's interest in STEM: A systematic methodological review*” examines how the use of a mixed methods approach enhances the comprehensiveness and robustness of an intervention design attempting to raise students' and girls' interest in STEM (Fàbregues et al.).

## Author contributions

MS wrote the draft version of the editorial. KU and SF made suggestions. All authors approved the submitted version.

